# Quality of life of families and siblings of children with cerebral palsy treated at a reference neurorehabilitation center in Brazil^✰^

**DOI:** 10.1016/j.jped.2024.03.010

**Published:** 2024-04-22

**Authors:** Bruno Leonardo Scofano Dias, Maura Calixto Cecherelli de Rodrigues, José Luiz Muniz Bandeira Duarte

**Affiliations:** a Rede SARAH de Hospitais de Reabilitação, Rio de Janeiro, RJ, Brazil; b Universidade do Estado do Rio de Janeiro (UERJ), Faculdade de Ciências Médicas, Programa de Pós-Graduação em Ciências Médicas, Rio de Janeiro, RJ, Brazil; c Universidade do Estado do Rio de Janeiro (UERJ), Faculdade de Ciências Médicas, Departamento de Pediatria, Rio de Janeiro, RJ, Brazil

**Keywords:** Quality of life, Family, Siblings, Cerebral palsy

## Abstract

**Objectives:**

To investigate the associations between caregivers’ burden, family quality of life (QoL), and siblings’ QoL in Brazilian families of children with cerebral palsy, and to analyze siblings’ QoL using as a parameter the QoL of typically developed Brazilian children.

**Methods:**

It was a cross-sectional study. The 212 families, 212 caregivers and 131 siblings completed the Family Quality of Life Scale, Burden Interview, and KIDSCREEN-27 Child and Adolescent Version and Parents Version questionnaires at a neurorehabilitation center in southeast Brazil. Univariable and multivariable models were used.

**Results:**

Family QoL significantly worsened as caregivers’ burden increased (95 % CI -0.66 to -0.38). Caregivers’ burden was significantly lower with increasing family QoL scores (95 % CI -0.52 to -0.30). Self-reported siblings’ QoL was significantly worse than that of their typically developed peers (95 % CI -7.6 to -3.6). Self-reported siblings’ QoL was significantly lower as siblings’ age (95 % CI -2.52 to -0.59) and caregivers’ burden (95 % CI -0.35 to -0.05) increased. Parent-reported siblings’ QoL was significantly lower with increasing caregivers’ burden (95 % CI -0.45 to -0.16) and higher as family QoL increased (95 % CI 0.09 to 0.37).

**Conclusions:**

The cross-sectional nature of these data precludes any statement of causality. Family QoL worsened with higher caregivers’ burden levels. Lower caregivers’ burden scores were associated with a higher family QoL. Siblings’ QoL was impaired as compared to typically developed peers, worse among older siblings, and as caregivers’ burden increased and better with higher family QoL levels. Future multicenter studies may validate the generalizability of the present findings.

## Introduction

Cerebral palsy (CP) is a major cause of childhood disability.^1^ Besides physical disability, clinical, neurological, orthopedic, sensory, mental health, and neurodevelopmental disorders may coexist and require continuous care. Consequently, there is a risk of burden on the main caregiver and an impact on the family and siblings’ quality of life (QoL). The QoL construct is multidimensional, covering the subjective appraisal of personal feelings, social relationships, local environment, societal values, and material conditions.^2^ Family QoL is a dynamic sense of well-being, collectively defined, and subjectively informed by its members.^3^ Caregivers’ burden represents the objective and subjective impairments associated with the care provided.^4^

Caregivers’ burden and their QoL in CP are well known and have been associated with patients’ gross motor impairments,^5^ intellectual disability, and emotional problems;^6^ caregivers’ mental^6^^,^^7^ and physical health;^7^ family coping patterns,^6^ income and composition;^6^^,^^7^ social support^7^ and school environment.^6^ Some studies correlated family functioning reported by caregivers (not specifically family QoL) with patients’ functional independence,^8^ and age;^9^ family composition, and income; parents’ education, and social integration.^8^^,^^9^ However, the family QoL concept involves the entire family system. This construct must be defined and informed by all family members, not just parents/caregivers. Although there is still a gap in the literature regarding its knowledge, some studies effectively studied family QoL in CP and identified low family education level,^10^ low environmental support^11^ and patient's severe physical and communication impairments^10^^,^^12^ as impact factors.

Caregivers’ burden may result in a reduction in the remaining attention time for other family members. Consequently, siblings may feel neglected and experience negative emotions.^13^ Only a few studies analyzed the life dimensions of siblings of children with CP such as psychological impacts,^14^^,^^15^ psychosocial issues,^16^^,^^17^ and QoL (poorer than that of controls in all three studies).^13^^,^^18^^,^^19^ However, none of these studies specifically and exclusively evaluated siblings’ QoL in CP and its correlations with influencing factors.

The authors aimed to study: (1) the associations between family QoL and caregivers’ burden in families of Brazilian children and adolescents with CP and its correlations with sociodemographic, functional, intellectual and clinical variables; (2) the QoL of siblings of Brazilian children and adolescents with CP using as a parameter the QoL of typically developed Brazilian children and adolescents and its correlations with sociodemographic, functional, intellectual and clinical variables, family QoL and caregivers’ burden. The hypotheses were: (1) family QoL and caregivers’ burden levels would be inversely associated, and worse sociodemographic, functional, intellectual, and clinical indicators would predict lower levels of family QoL and higher levels of caregivers’ burden; (2) siblings’ QoL levels would be lower than that of typically developed Brazilian children and adolescents, and worse sociodemographic, functional, intellectual, clinical, family QoL and caregivers’ burden indicators would predict poorer levels of siblings’ QoL.

## Method

The study used a cross-sectional design.

As the authors do not have reliable descriptions in the literature about the prevalence of CP in Brazil and in the city of Rio de Janeiro, it was not possible to carry out the sample size calculation and we chose to use a convenience sample. Participants were recruited from a sample of children and adolescents with CP consecutively treated at a neurorehabilitation center in southeast Brazil. The following inclusion criteria were used: families of children and adolescents with CP aged between 2 and 16 years with typically developed siblings aged between 10 and 15 years. The age range of the patients was chosen so that the authors could clearly define the functional classification of each of them. The age range of the siblings was chosen so that it would be exactly matched with the age range of the sample of typically developed Brazilian children and adolescents with whom their QoL would be compared. Families and siblings would be excluded if they did not return completed instruments for measuring family QoL, caregiver burden, siblings’ QoL, and family economic classification. The study was approved by the Research Ethics Committee of the SARAH Network of Rehabilitation Hospitals (Certificate of Submission for Ethics Assessment 50381121.1.0000.0022). Data was collected between November 2021 and November 2022.

The authors followed a pre-established structured protocol. Patients’ legal guardians and siblings were approached by the researcher. The objectives of the study were explained. Legal guardians and siblings completed informed consent for the research and publication of results. Families, caregivers and siblings completed the instruments for measuring family QoL, caregiver burden, siblings’ QoL, and family economic classification in their homes and returned them to the researchers at the institution. Other demographic, functional, intellectual and clinical data on patients, and sociodemographic data on families and on siblings were collected from the patients' electronic medical records. Two hundred and twelve families and 131 siblings were included. All families and siblings returned completed questionnaires.

### Measures

#### Family quality of life

The Family Quality of Life Scale (FQoLS) questionnaire was used to analyze family QoL. This instrument was designed to evaluate QoL in families with children with disabilities. Responses vary from 1 to 5 in each of the 25 items. The total score is obtained through the sum of all items.^20^ Satisfaction levels are classified as very dissatisfied (25 to 44 points), dissatisfied (45 to 64 points), moderately satisfied (65 to 84 points), satisfied (85 to 104 points), and very satisfied (105 to 125 points). FQoL was defined as follows: low family QoL (very dissatisfied, dissatisfied, moderately satisfied) / high family QoL (satisfied and very satisfied).

#### Caregivers’ burden

Caregivers’ burden was assessed using the Burden Interview (BI). This instrument assesses caregivers’ burden associated with functional and behavioral aspects of patients with disabilities. Responses vary from zero to 4 in each of the 22 items. The total score is obtained through the sum of all items. Burden levels are classified as absent (≤ 21 points), mild to moderate (22 to 40 points), moderate to severe (41 to 60 points), and severe (≥ 61 points).^21^ Caregivers’ burden was defined as follows: low caregivers’ burden (absent and mild to moderate) / high caregivers’ burden (moderate to severe and severe).

#### Siblings’ quality of life

Siblings’ QoL was evaluated using the self-report and parent-proxy versions of the generic KIDSCREEN measure. KIDSCREEN (versions with 52, 27 and 10 items) is designed for 8 to 18 years olds. The authors used the KIDSCREEN-27 Child and Adolescent Version (KIDSCREEN-27 CAV) and Parent Version (KIDSCREEN-27 PAV). These versions comprise 27 items distributed in five domains: physical well-being; psychological well-being; autonomy and relationship with parents; social support and peers; and school environment. Scores are coded from 1 to 5. Raw scores are obtained by summing items in each QoL domain. Rasch person parameters are then allocated to each score and transformed to a zero to 100 scale.^22^^,^^23^^,^^24^

#### Quality of life of typically developed Brazilian children and adolescents

The authors used the data described by Farias et al. (2017)^25^ as a QoL parameter for typically developed Brazilian children and adolescents. In this study, the authors used a sample of individuals aged 10 to 15 years, for reproducibility, internal consistency and construct validity of the KIDSCREEN-27 in Brazil.

#### Family economic classification and educational level

Family economic classification and educational level were assessed using the Brazil Criteria. This criterion is a scoring system for housing conditions, consumer goods, access to public services and family main educational level of Brazilian families. The sum of the scores of all items classifies families into descending economic classes A, B, C, D and E.^26^ Family economic classification and educational level were defined respectively as follows: high-income family (economy classes A and B) / low-income family (economy classes C and D/E); low educational level (illiterate to incomplete high school) / high educational level (high school to complete undergraduate education).

#### Patients’ functional gross motor and swallowing capacities

Patients’ functional gross motor capacities were evaluated using the Gross Motor Function Classification System (GMFCS). The goal of the GMFCS is to classify the child's gross motor function through five increasing severity motor levels (I to V), characterizing the child's motor performance.^27^

The swallowing capacities were analyzed using the Eating and Drinking Ability Classification System (EDACS). EDACS is an instrument created to measure the safety and efficiency of swallowing conditions specifically in children with CP. Its numerical classification system comprises a scale from I to V, with the higher the score, the worse the swallowing condition.^28^

Patients’ functional gross motor and swallowing capacities were respectively defined as follows: low motor impairment (GMFCS I, II, III) / high motor impairment (GMFCS IV and V); low swallowing impairment (EDACS I, II, III) / high swallowing impairment (EDACS IV and V).

#### Patients’ intellectual impairment and epilepsy

Intellectual impairment was defined as the presence of global developmental delay or intellectual disability analyzed by the Vineland Adaptive Behavior Scales (VABS). VABS is the most validated instrument for adaptive functioning following brain pathology in children in low- and middle-income countries.^29^

Epilepsy was classified as absent or present using the definition of the International League Against Epilepsy as a disease of the brain defined by any of the following conditions: at least two unprovoked (or reflex) seizures occurring more than 24 h apart; one unprovoked (or reflex) seizure and a probability of further seizures similar to the general recurrence risk (at least 60 %) after two unprovoked seizures, occurring over the next 10 years; diagnosis of an epilepsy syndrome.^30^

FQoLS,^20^ BI,^21^ KIDSCREEN-27,^25^ GMFCS,^27^ EDACS^28^ and VABS^29^ are translated, validated and adapted for the Portuguese language spoken in Brazil and for Brazilian culture.

### Statistical analysis

Data were summarized using mean (standard deviation [SD]) or median (interquartile range [IQR]) for continuous variables and frequency (percent) for categorical variables. There were no missing data. Variable distributions were visualized and quality-checked with bar and violin plots.

Comparisons of domains and total scores on KIDSCREEN-27 CAV of siblings and typically developed Brazilian children and adolescents, and comparisons of domains and total scores on KIDSCREEN-27 CAV and KIDSCREEN-27 PAV of siblings were performed using *t*-test (parametric) and Wilcoxon test (non-parametric) respectively for samples with and without normal distribution. Effect sizes and 95 % confidence intervals (CI) of each of these comparisons were calculated.

Multivariable models were constructed for FQoLS, BI, KIDSCREEN-27 CAV, and KIDSCREEN-27 PAV total scores. The authors checked for linearity, normality, collinearity, auto-correlation and homoscedasticity. Estimates were presented with 95 % CI. Two-sided p-values less than 0.05 were considered significant. All statistical analyses were performed in Jamovi 2.3.16 (Jamovi, 2022). STROBE reporting guidelines were followed.

## Results

### Sample characteristics

#### Families and caregivers

There were 212 families, mostly with two legal guardians, low income, high educational level, and high family QoL (77.4 %). The median (IQR) of the FQoLS total score was 96.0 (86.0–107.0). The 212 caregivers were female, with a predominantly low caregiver burden (78.7 %). The median (IQR) of the BI total score was 30.0 (22.0–38.2) (Table 1).

**Table 1:** Characteristics of families, caregivers, patients and siblings.

Families N (%).	212 (100)
Two legal guardians	166 (78.3)
One legal guardian	46 (21.7)
Low-income	.167 (78.7)
High-income	45 (21.3)
Low educational level	77 (36.3)
High educational level	135 (63.7)
Very dissatisfied with family QoL	1 (0.5)
Dissatisfied with family QoL	2 (0.9)
Moderately satisfied with family QoL	45 (21.2)
Satisfied with family QoL	100 (47.2)
Very satisfied with family QoL	64 (30.2)

**Family Quality of Life Scale total score Median (IQR)**	96.0 (86.0–107.0)
**Caregivers’ burden N (%)**	**212 (100)**

Absent burden	45 (21.2)
Mild to moderate burden	122 (57.5)
Moderate to severe burden	38 (17.9)
Severe burden	7 (3.3)
**Burden Interview total score Median (IQR)**	30.0 (22.0–38.2)

**Patients (N)**	**212**

**Age N (%)**	
2–5 years	93 (43.9)
6–9 years	74 (34.9)
10–12 years	28 (13.2)
13–16 years	17 (8.0)
**Age (years) Median (IQR)**	**6 (4–10)**
**Sex N (%)**	
Male	120 (56.6)
Female	92 (43.4)
**Functional characteristics N (%)**	
Low motor impairment	109 (51.4)
High motor impairment	103 (48.6)
Low swallowing impairment	182 (85.9)
High swallowing impairment	30 (14.1)
**Intellectual impairment N (%)**	
Present global developmental delay or intellectual disability	169 (79.7)
Absent global developmental delay or intellectual disability	43 (20.3)
**Epilepsy N (%)**	
Present epilepsy	108 (50.9)
Absent epilepsy	104 (49.1)

**Siblings (N)**	**131**

**Age N (%)**	
10–12 years	65 (49.6)
13–15 years	66 (50.4)
**Age (years) Median (IQR)**	13 (11–14)
**Sex N (%)**	
Male	62 (47.3)
Female	69 (52.7)

IQR, interquartile range; N, number;%, percentage.

#### Patients, siblings and typically developed Brazilian children and adolescents

There were 212 patients (mean age 6 years 10 months, SD 3 years 6 months; median age 6 years, IQR 4 years-10 years; 93 aged 2 to 5 years; 120 males), mostly with intellectual impact, low motor and swallowing impairments and epilepsy. The authors assessed 131 siblings (mean age 12 years 6 months, SD 1 year 8 months; median age 13 years, IQR 11 years-14 years; 66 aged 13 to 15 years; 69 females). These characteristics are summarized in Table 1.

The sample of typically developed Brazilian children and adolescents comprised 1321 individuals (752 aged 10 to 12 years; 709 females), mostly from low-income families.

### Univariable models

#### Self-reported quality of life of siblings and typically developed Brazilian children and adolescents

Siblings’ self-reported QoL (KIDSCREEN-27 CAV domains and total mean scores) were significantly lower than that of typically developed Brazilian children and adolescents (KIDSCREEN-27 CAV domains and total mean scores), as follows: physical well-being (*p* < .001; 95 % CI −9.1 to −3.1; small effect size), psychological well-being (*p* < .001; 95 % CI −10.0 to −4.3; medium effect size), autonomy and relationship with parents (*p* = .006; 95 % CI −5.6 to −1.3; small effect size), social support and peers (*p* = .04; 95 % CI −6.9 to −1.9; small effect size), school environment (*p* < .001; 95 % CI −10.5 to −3.0; small effect size) and total score (*p* < .001; 95 % CI −7.6 to −3.6; medium effect size) (Table 2).

**Table 2:** Comparison of KIDSCREEN-27 Child and Adolescent Version mean scores between siblings and typically developed Brazilian children and adolescents.

		**KIDSCREEN-27 Child and Adolescent Version**					
		**Siblings**	**TDBCA**		**95 % CI**		
	***N*=131**	**Mean (SD)**	**Mean (SD)**	***P***	**Inferior**	**Superior**	**Effect size**
Physical well-being		70.6 (16.3)	77.7 (14.1)	**<0.001**	- 9.1	- 3.1	- 0.389
Psychological well-being		78.5 (13.9)	85.7 (12.1)	**<0.001**	- 10.0	- 4.3	- 0.661
Autonomy and relationship with parents		72.3 (14.1)	77.0 (15.8)	**0.006**	- 5.6	- 1.3	- 0.274
Social support and peers		76.7 (17.9)	81.9 (16.5)	**0.04**	- 6.9	- 1.9	- 0.207
School environment		78.0 (16.2)	85.5 (14.5)	**<0.001**	- 10.5	- 3.0	- 0.482
Total score		75.2 (10.9)	81.3 (10.5)	**<0.001**	- 7.6	- 3.6	- 0.550

Bold type indicates statistical significance.

CI, confidence interval; N, number; TDBCA, typically developed Brazilian children and adolescents.

#### Self-reported and parent-reported quality of life of siblings

There were no significant differences between self- and parent-reported siblings’ QoL (respectively KIDSCREEN-27 CAV and KIDSCREEN-27 PAV domains and total median scores), except for the social support and peers domain, in which siblings’ perception was significantly more positive (*p* = .002; 95 % CI 5.0 to 15.0) (Fig. 1, supplementary material).

### Multivariable models

#### Family quality of life and caregivers’ burden multivariable models

Family QoL (FQoLS total scores) was significantly lower as caregivers’ burden (BI total scores) increased (95% CI −0.66 to −0.38) and significantly higher in families with low educational levels (95% CI 0.38 to 8.16).

Caregivers’ burden on BI total scores was significantly lower with increasing levels of family QoL (FQoLS total scores) (95% CI −0.52 to −0.30) (Table 3).

**Table 3:** Estimated regression coefficients for variables included in multivariable models for Family Quality of Life Scale and Burden Interview total scores.

Characteristic	FQoLS total scores			BI total scores		
	CF	95 % CI	*P*	CF	95 % CI	*P*
**Patients’ age**	0.24	−0.22,0.71	0.30	−0.38	−0.80,0.02	0.064
**Patients’ sex, male vs female**	1.37	−2.19,4.93	0.44	−1.03	−2.14,4.22	0.521
**GMFCS, low impairment vs high impairment**	−0.25	−4.43,3.91	0.90	0.66	−3.06,4.38	0.727
**EDACS, low impairment vs high impairment**	3.73	−1.83,9.31	0.18	−0.69	−569,4.30	0.784
**GDD/ID, absent vs present**	0.371	−4.64,5.38	0.88	3.00	−1.46,7.46	0.186
**Epilepsy, absent vs present**	−0.82	−4.89,3.24	0.68	1.82	−1.80,5.45	0.323
**Family Composition, 2 legal guardians vs 1 legal guardian**	−1.79	−6.12,2.54	0.41	1.78	−2.08,5.65	0.363
**Family Economic Level, low-income vs high-income**	−3.46	−7.92, 0.99	0.12	0.679	−3.32,4.68	0.738
**Educational Level, low level vs high level**	4.27	0.38,8.16	**0.03**	2.50	−0.99,6.00	0.159
**BI total score**	−5.52	−0.66,−0.38	**<0.001**	–	–	–
**FQoLS total score**	–	–	–	−0.41	−0.52,−0.30	**<0.001**

Bold type indicates statistical significance.

Variables not included in the multivariable models are indicated with a dash.

Abbreviations: BI, Burden Interview; CF, coefficient; CI, confidence interval; CP, cerebral palsy; EDACS, Eating and Drinking Ability Classification System; FQoLS, Family Quality of Life Scale; GDD, global developmental delay; GMFCS, Gross Motor Function Classification System; ID, intellectual disability.

#### Siblings’ multivariable models

Self-reported siblings’ QoL (KIDSCREEN-27 CAV total scores) was significantly lower as siblings’ age (95% CI −2.52 to −0.59) and caregivers’ burden (BI total scores) (95% CI −0.35 to −0.05) increased, and in families with two legal guardians (95% CI −9.49 to −0.29).

Parent-reported siblings’ QoL (KIDSCREEN-27 PAV total scores) was significantly lower with increasing levels of caregivers’ burden (BI total scores) (95% CI −0.45 to −0.16) and significantly higher as family QoL (FQoLS total scores) increased (95% CI 0.09 to 0.37) (Table 4).

**Table 4:** Estimated regression coefficients for variables included in multivariable models for siblings’ KIDSCREEN-27 Child and Adolescent and Parent Versions total scores.

Characteristic	KIDSCREEN-27 CAV total scores			KIDSCREEN-27 PAV total scores		
	CF	95 % CI	*P*	CF	95 % CI	*P*
**Siblings’ age**	−1.56	−2.52, −0.59	**0.002**	−0.76	−1.68, 0.15	0.101
**Siblings’ sex, male vs female**	−3.48	−6.97,6.24	0.05	1.95	−1.37, 5.28	0.248
**Patients’ age**	−0.30	−0.80, 0.19	0.22	0.05	−0.42, 0.53	0.814
**Patients’ sex, male vs female**	−2.57	−6.23, 1.08	0.16	−1.35	−4.85, 2.13	0.442
**GMFCS, low impairment vs high impairment**	0.24	−3.70, 4.20	0.90	−2.13	−5.91, 1.63	0.265
**EDACS, low impairment vs high impairment**	1.34	−4.81, 7.51	0.66	−1.45	−7.34, 4.42	0.624
**GDD/ID, absent vs present**	3.14	−1.52, 7.81	0.18	2.38	−2.07, 6.84	0.291
**Epilepsy, absent vs present**	−0.32	−4.09, 3.44	0.86	3.01	−0.57, 6.61	0.099
**Family composition, 2 legal guardians vs 1 legal guardian**	−4.89	−9.49, −0.29	**0.03**	−3.39	−7.78, 0.99	0.129
**Family Economic Classification, low-income vs high-income**	−0.73	−5.10, 3.63	0.74	−0.30	−4.48, 3.86	0.884
**Educational Level, low level vs high level**	−0.11	−3.98, 3.75	0.95	−1.85	−5.55, 1.83	0.321
**BI total score**	−0.20	−0.35, −0.05	**0.007**	−0.30	−0.45, −0.16	**< .001**
**FQoLS total score**	0.14	−0.01, 0.29	0.06	0.23	0.09, 0.37	**0.002**

Bold type indicates statistical significance.

Abbreviations: BI, Burden Interview; CF, coefficient; CI, confidence interval; CP, cerebral palsy; EDACS, Eating and Drinking Ability Classification System; FQoLS, Family Quality of Life Scale; GDD, global developmental delay; GMFCS, Gross Motor Function Classification System; ID, intellectual disability; KIDSCREEN-27 CAV, KIDSCREEN-27 Child and Adolescent Version; KIDSCREEN-27 PA, KIDSCREEN-27 Parents Version.

## Discussion

To the best of our knowledge, the present study is the first to specifically analyze the associations between family QoL, caregivers’ burden, and siblings’ QoL in families of children and adolescents with CP. Remarkably, and in disagreement with our hypotheses, patients’ functional, intellectual and clinical variables were not significantly correlated with family QoL, caregivers’ burden the self- and parent-reported siblings’ QoL. In accordance with the present hypotheses, family QoL and caregivers' burden were important significant influencing factors for each other and siblings had significantly worse QoL than that of typically developed Brazilian children and adolescents. Family QoL was significantly worse in those with higher caregivers’ burdens. Caregivers’ burden was significantly lower as patients’ age and family QoL increased. Self-reported siblings’ QoL was significantly lower with increasing siblings’ age and caregivers’ burden, and parent-reported siblings’ QoL was lower and higher respectively as caregivers’ burden and family QoL increased.

In the present sample, most caregivers expressed a low burden, and families declared mostly a high family QoL. In contradiction, Sogbossi et al. found CP families with high impacts on family QoL in Benin^10^. Other studies did not evidence significant family QoL differences between CP families and those with typically developed controls in Croatia^11^ and families with children with different developmental diagnoses in Israel.^12^ These differences and similarities could possibly be explained by socioeconomic differences between high (Croatia and Israel) / upper middle-income (Brazil) and low-income (Benin) countries.

The authors found family QoL significantly higher and lower respectively in families with low educational levels and with increasing caregivers’ burden scores. Probably, a low educational level generated lower expectations with family QoL, and a high caregivers’ burden reduced their time and attention for the family. A study performed in Bosnia and Herzegovina also observed significantly better family QoL in families of children with CP with low educational levels.^5^ Patients’ functional, intellectual and clinical variables were not significantly correlated with family QoL in the sample. Once again, Sogbossi et al. found contrary findings, with family QoL negatively correlated with increasing GMFCS levels.^10^ The present results showed caregivers’ burden was significantly lower as family QoL increased, and not significantly associated with patients’ functional, intellectual and clinical variables and family socioeconomic factors.

The samples of siblings of children and adolescents with CP and typically developed Brazilian children and adolescents were age-matched (10 to 15 years), and showed comparable frequencies in age groups, gender and socioeconomic characteristics. Siblings experienced significantly poorer QoL in relation to typically developed Brazilian children and adolescents. Dinleyici et al. also found siblings’ QoL in CP significantly lower than that of controls in Turkey.^13^ Rana et al. correlated siblings’ QoL with that of their brothers and sisters with CP in India, but siblings’ QoL was not compared with that of typically developed children.^18^ Aran et al. described lower mean QoL scores in siblings of children with CP than in peers of the same age in Israel. However, such mean scores were not compared to assess whether the differences were significant.^19^

Siblings’ scores in all domains of the KIDSCREEN-27 CAV were significantly lower than those of typically developed Brazilian children and adolescents, however, these differences showed small effect sizes, except for psychological well-being, with a medium effect size. In agreement with the present results, Dinleyici et al. observed the psychosocial health of siblings of children with CP was significantly lower than that of controls.^13^ Hugarian^14^ and Israeli^15^ studies found mental illness more common in siblings of children with CP than in the general population.

The authors found no significant discrepancy between self-reported and parent-reported siblings’ QoL, except for the social support and peers domain on KIDSCREEN-27, in which siblings' perception was significantly more positive. This finding could be explained by an overconfidence of siblings in their peers and a more pessimistic opinion of their parents. In contrast, Dinleyici et al. found a worse self-reported than parent-reported siblings’ QoL.^13^

The present results showed self-reported siblings’ QoL significantly worse with increasing siblings’ age and caregivers’ burden and in families with two legal guardians. Possibly, with increasing age and maturity, even with two guardians present at home, the siblings have realized the impact of caregivers’ burden, therefore affecting their QoL.

Parent-reported siblings’ QoL was lower and higher respectively as caregivers’ burden and family QoL increased. It is possible that parents felt that, regardless of other characteristics, the only influences on siblings’ QoL were how much the caregiver was burdened and how the family functioned.

These results show that family QoL and caregivers’ burden might be extremely important for the QoL of siblings of children with CP. Given the mutual influences between individuals in a family, siblings’ QoL could also be influenced by and influence CP patients’ QoL, hypotheses yet to be studied.

Patients’ functional, intellectual and clinical variables were not significantly correlated with self- and parent-reported siblings’ QoL in this sample. Aran et al. (2007) also evidenced no correlations between siblings’ QoL and patients’ GMFCS levels.^19^

This was a single-center study, with a convenience sample, therefore, the authors cannot generalize these findings. The cross-sectional nature of the available data precludes any statement of causality. The literature gap regarding studies on family QoL and siblings' QoL in families with children with CP, the varied methodologies used in these studies and the socioeconomic and cultural differences between the countries where they were carried out, reduced the possibilities of comparisons, so the authors had to focus mainly on the interpretation of our own results. These are the main limitations. The strength of the study is its innovative character in investigating the associations between caregivers’ burden, family QoL, and siblings’ QoL in families of children with CP and its correlations with several variables.

Considering CP as a major cause of childhood disability, the present results reinforce the relevance of knowing the factors that influence caregivers’ burden, family QoL, and siblings’ QoL in CP, and of further research in these fields of knowledge, providing secure bases for the construction of supporting programs for the whole family, aimed at reducing caregivers' burden, improving family QoL, and meeting the individual needs of the siblings. Future multicenter studies may validate the generalizability of these findings.

## Conflicts of interest

The authors declare no conflicts of interest.

## Appendix Supplementary materials

Image, application 1
