# Supplementary material for: Quality of life of families and siblings of children with cerebral palsy treated at a reference neurorehabilitation center in Brazil
Source: J Pediatr (Rio J). 2024 Apr 22;100(5):519–26. doi: 10.1016/j.jped.2024.03.010 (PMC11361884; doi:10.1016/j.jped.2024.03.010)
Supplement: Supplementary file 1 [file mmc1.docx]

**JPED-D-23-00404_Supplementary Material**

Supplementary material

**
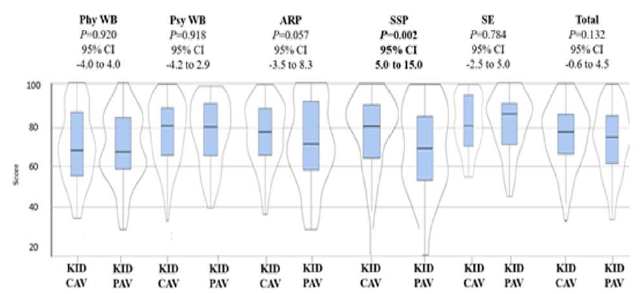
**

**Figure 1. KIDSCREEN-27 Child and Adolescent Version and Parent Version domains and total median scores**

Bold type indicates statistical significance. ARP, autonomy and relationship with parents; CI, confidence interval; KIDCAV, KIDSCREEN-27 Child and Adolescent Version; KIDPAV, KIDSCREEN-27 Parent Version; Phy WB, physical well-being; Psy WB, psychological well-being; SE, school environment; SSP, social support and peers.
